# Supplementary material for: What does it take to learn the rules of RNA base pairing? A lot less than you may think
Source: bioRxiv. 2025 Aug 2:2025.07.31.668042. Preprint. [Version 1] doi: 10.1101/2025.07.31.668042 (PMC12324431; doi:10.1101/2025.07.31.668042)
Supplement: Supplement 1 [file media-1.gz › supplemental_material/experiments/g6_optimize_param_conus_rnabench_RNaseP.25.B_uniform/losses_i3.pdf]

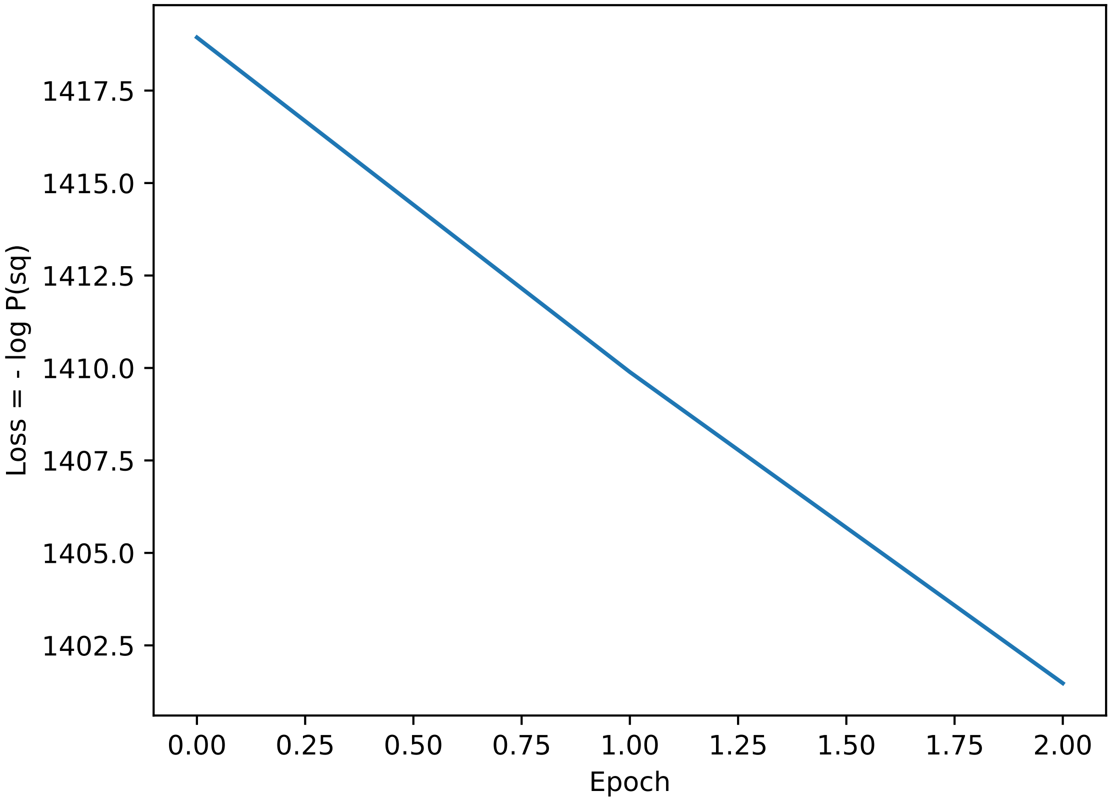

Loss = - log P(sq)
1417.5
1415.0
1412.5
1410.0
1407.5
1405.0
1402.5
0.00
0.25
0.50
0.75
1.00
1.25
1.50
1.75
2.00
Epoch
